# Millet manuring as a driving force for the Late Neolithic agricultural expansion of north China

**DOI:** 10.1038/s41598-018-23315-4

**Published:** 2018-04-03

**Authors:** Xin Wang, Benjamin T. Fuller, Pengcheng Zhang, Songmei Hu, Yaowu Hu, Xue Shang

**Affiliations:** 1 Key Laboratory of Vertebrate Evolution and Human Origins of Chinese Academy of Sciences, Institute of Vertebrate Paleontology and Paleoanthropology, Chinese Academy of Sciences, Beijing, 100044 China; 2 Department of Archaeology and Anthropology, University of Chinese Academy of Sciences, Beijing, 100049 China; 3 Department of Archaeology and Heritage Studies, School of Culture and Society, Aarhus University, Moesgård Allé 20, DK-8270 Højbjerg, Denmark; 4 Shaanxi Provincial Institute of Archaeology, Xi’an, 710043 China

## Abstract

Research in to the nature of Neolithic agriculture in China is often focused on topics such as the domestication and spread of cereal crops and the reconstruction of human and animal diets in the past. Field management practices, such as organic manuring, have not been systematically investigated in Chinese archaeology. Here we present an isotopic dataset for archaeological foxtail millet (*Setaria italica*) and common millet (*Panicum miliaceum*) grains as well as associated faunal remains (both domesticated and wild) from seven sites in the Baishui Valley of north China, in order to find direct evidence of organic manuring during the Late Neolithic period. The elevated nitrogen isotope values of the millet grains (5500-3500 cal BP) in comparison with the estimated local vegetation indicates that millets were organically manured by animal dung, mostly likely originating from domestic pigs. Considering the low nitrogen contents of loess soils and their unsuitability for intensive cultivation, this organic manuring by animal dung would have played a key role in maintaining soil productivity and crop yield, which was necessary to support the demands of agriculture and cultural expansion during the Late Neolithic on the Loess Plateau of China.

## Introduction

Long-term farming practices where crops are annually cultivated in the same location can result in the depletion of soil nutrients and a loss of crop yields and quality^1^. However, to counter this degradation of soil nutrients, farmers have recognized that the addition of animal dung (organic manuring) can regenerate soil fertility and maintain high crop productions. Recently, there has been a dramatic increase in the stable isotope ratio analysis of modern and archaeological plant remains to infer environmental conditions and crop management practices such as manuring^2–12^. In general, the addition of organic manure (animal waste) to crops results in an increase in soil δ^15^N values^13,14^, and plants grown in these soils will have elevated δ^15^N values in comparison to those grown in natural or unmanured soils^5,8^. Based on the above principle, elevated δ^15^N values of charred cereals have been used as good indicators of past manuring practices at a number of archaeological sites in Europe^8,10,15–19^.

Foxtail millet (*Setaria italica*) and common millet (*Panicumm miliaceum*), are believed to have been domesticated in northern China around 10000 BP^20–23^. Millet sites are located sporadically along the eastern edge of the Loess Plateau around 8000 BP, and by c. 6000 BP, quantitative charred millet remains are found at numerous sites in the Yellow River Valleys^24–27^. From approximately 7000 to 5000 BP, the Yangshao culture became the dominate culture in northern China. This culture had a deep and significant influence on later cultures and established a foundation for the development of the Chinese civilization^28^. While the farming practices of the Yangshao culture have been studied in the past^29–31^, only limited research has focused on the isotopic analysis of millets from Chinese archaeological sites^32–34^. None of these past studies examined manuring practices during the Chinese Neolithic. Here we report radiocarbon dates (n = 10) and carbon (δ^13^C) and nitrogen (δ^15^N) stable isotope ratio results for charred foxtail (n = 29) and common (n = 20) millet grains from seven Yangshao archaeological sites (Xiahe, Mapo, Nanshantou, Beishantou, Xishan, Hanzhai, Muwanghe) in the Baishui Valley of Shaanxi Province. Associated fauna (n = 42) from two sites (Xiahe and Mapo) are also isotopically examined to investigate animal husbandry practices. The focus of this research is to investigate if manuring was practiced during the Late Chinese Neolithic, and if so, how this important farming practice contributed to the agricultural expansion and population growth of the Yangshao culture.

## Archaeological context

**Figure 1:**
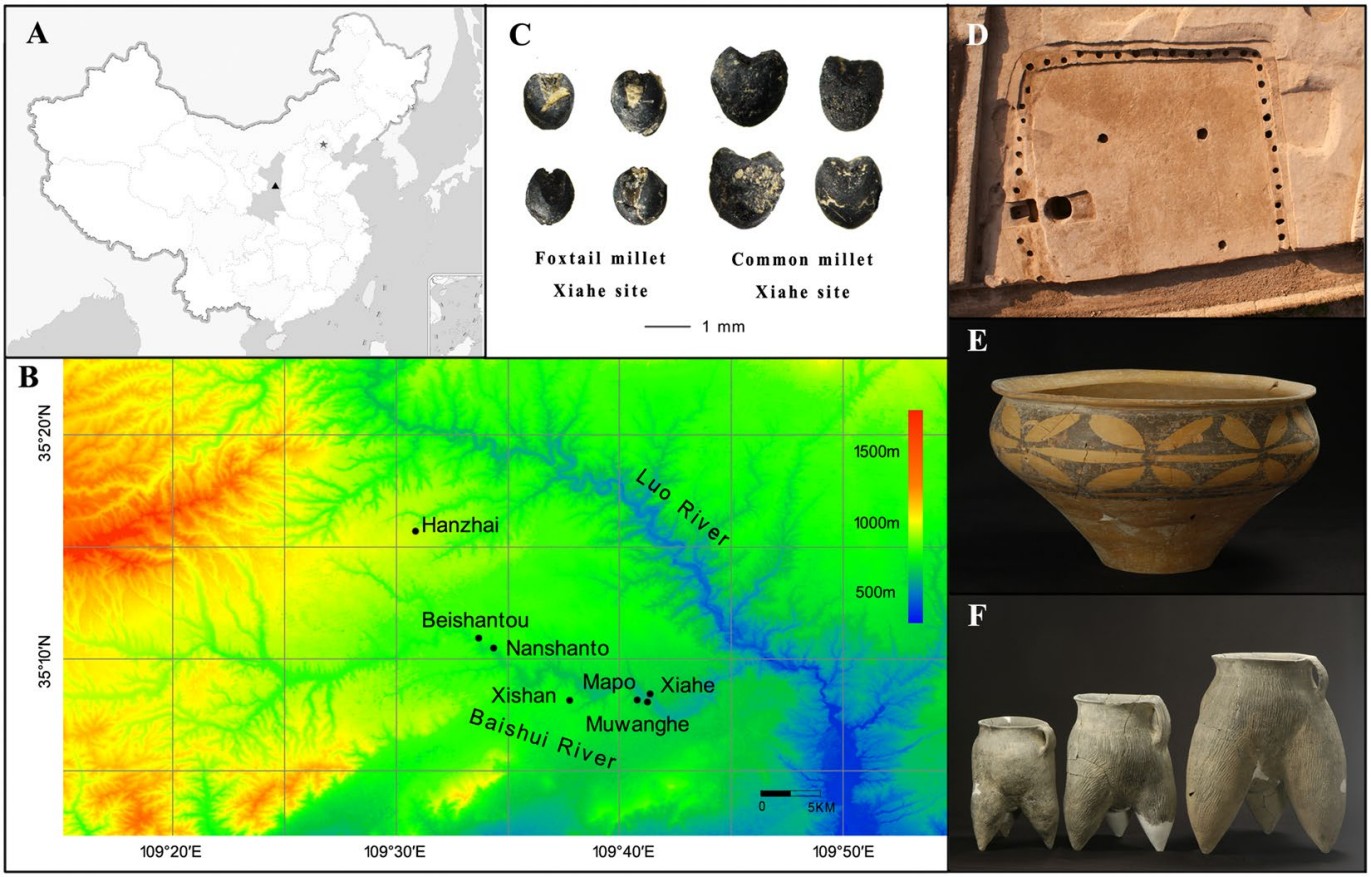
(**A**,**B**) Maps showing the locations of the five Baishui Valley sites, Shaanxi Province, China. (**C**) Charred foxtail and common millet grains from the Xiahe site. (**D**) Aerial view of the excavation of a pentagon shaped house at Xiahe. (**E**,**F**) Samples of typical pottery that was recovered from Xiahe. Maps were created with software Global Mapper v 11 (http://www.bluemarblegeo.com/products/global-mapper.php). Photograph reproduced with the permission of excavator of Xiahe site.

The Baishui River is one of the tributaries of the Yellow River. It is located in the southern part of the Loess Plateau, where a large number of Neolithic agricultural sites have been discovered. During 2010–2012, excavations in the region by a team from the Shaanxi Provincial Institute of Archaeology, and abundant archaeobotanical and faunal remains were recovered at seven archeological sites, and these provided a good collection for studying the crop management and animal husbandry practices in northern China from the late-Yangshao to Longshan cultural periods (Fig. 1).

All seven sites date to the late-Neolithic, spanning the cultures of the: Yangshao, Miaodigou II and Longshan (Table S1). The Xiahe site was inhabited from the middle Yangshao culture to the Longshan culture. Nine houses, 79 urn burials, 25 adult burials, and 97 pits were excavated in an area of 1700 m^2^. The houses are south facing, semi-subterranean and were pentagon shaped (Fig. 1D). House F1 was the largest, occupying 365 m^2^, and 36 postholes were identified. It is speculated that house F1 could have been a sacrificial or assembly hall. The large scale of the house foundations at Xiahe is rare for the Yangshao period, and it was suggested that this site could have been an origin for palace architecture in China^35^. Additional artifacts included: pottery, stone artifacts, and plant and animal remains (Fig. 1E,F).

At the Mapo site 11 houses, a kiln and a pit were identified. Pit H1 was excavated in 2012 and 215 potteries were identified characteristic of the late Miaodigou II culture, and it was rich in archaeobotanical and animal remains. At the Nanshantou site a house, kiln, two ditches, 16 pits and numerous potteries were recovered. Artifacts of the Dongzhuang type are notable at the Nanshantou site, which represents a transitional period from Banpo to the Miaodigou cultures^36^. At Xishan two pits were excavated and the recovered pottery belongs to the Yangshao culture. Two pits were also excavated at Hanzhai and these date to the Miaodigou II culture.

## Results

### Radiocarbon dating

The radiocarbon ages for the foxtail and common millets from the seven sites are listed in Table 1. According to the data, the sites date from 5575 to 4070 cal BP. These results are in general agreement with the rough dates inferred from the analysis of pottery typology in this region. In total, the sites date to three periods of the Late Neolithic: the late-Yangshao period (c. 5500-4300 BP), the Miaodigou II period (4300-4100 BP) and the Longshan period (c. 4100-3500 BP).

**Table 1:** Radiocarbon ages from the Baishui Valley sites.

Lab Code	Sample type	Site	Unit	AMS ^14^C Age (yr BP)	Cal Radiocarbon Age (2σ range, cal yr BP)
BA130786	Charcoal	Xiahe	H43	4515 ± 20	5050–5190
BA130787	Foxtail millets	Xiahe	H20②	3770 ± 30	4070–4240
Beta-427728	Foxtail millets	Xiahe	H45	3680 ± 30	4250–4425
BA130789	Foxtail millets	Mapo	H1	3820 ± 25	4140–4300
BA130921	Common millets	Nanshantou	F2	4685 ± 30	5310–5480
BA130922	Foxtail millets	Nanshantou	H15	4340 ± 30	4840–4980
BA130790	Foxtail millets	Nanshantou	H10	4265 ± 25	4820–4865
BA131926	Foxtail/Common millets	Xishan	H5	4305 ± 40	4820–4980
BA131927	Foxtail millets	Hanzhai	H2	3925 ± 25	4250–4440
Beta-422852	Foxtail millets	Beishantou	H1	4410 ± 30	5320–5575

BA = Peking University.

### Isotopic results of millet grains

**Figure 2:**
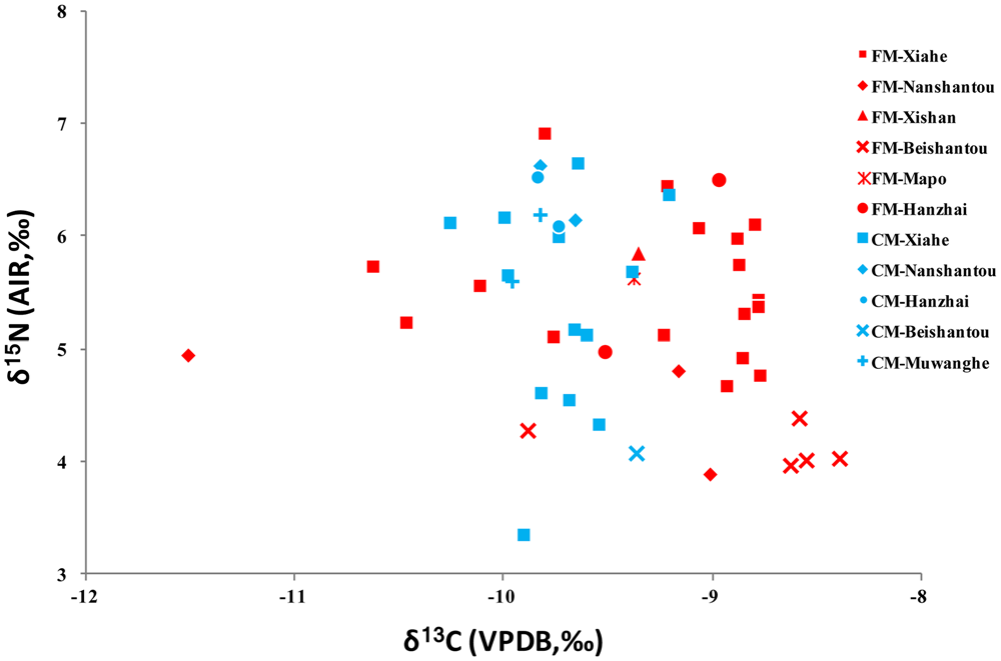
δ^13^C and δ^15^N results from millet grains from late Neolithic sites located in the Baishui Valley. (FM = Foxtail millet, CM = Common millet).

Figure 2 shows the δ^13^C and δ^15^N results of the foxtail and common millets from the sites, and Table S2 summarizes the corresponding results. In general, the foxtail millet has a wider isotopic range than the common millet in terms of δ^13^C values while the δ^15^N values are similar (Fig. 2). The δ^13^C values of the foxtail millet range from −11.5‰ to −8.4‰ (mean: −9.3 ± 0.7‰, n = 29), while those of the common millet range from −10.3‰ to −9.2‰ (mean: −9.7 ± 0.2‰, n = 20). The mean value of the foxtail millet is significantly different from that of the common millet (ANOVA, *p* = 0.008), and this appears related to physiological differences between the species^33^. The δ^15^N values of the foxtail millet grains range from 3.9‰ to 6.9‰ (5.2 ± 0.8‰, n = 29), and the δ^15^N values of the common millet range from 3.3‰ to 6.6‰ (5.5 ± 0.9‰, n = 20). The foxtail (5.2 ± 0.8‰) and common millet (5.5 ± 0.9‰) display nearly identical δ^15^N mean ± SD values (Fig. 2, ANOVA, *p* = 0.218). This shows that the δ^15^N values of the foxtail and common millets are indistinguishable from each other.

**Figure 3:**
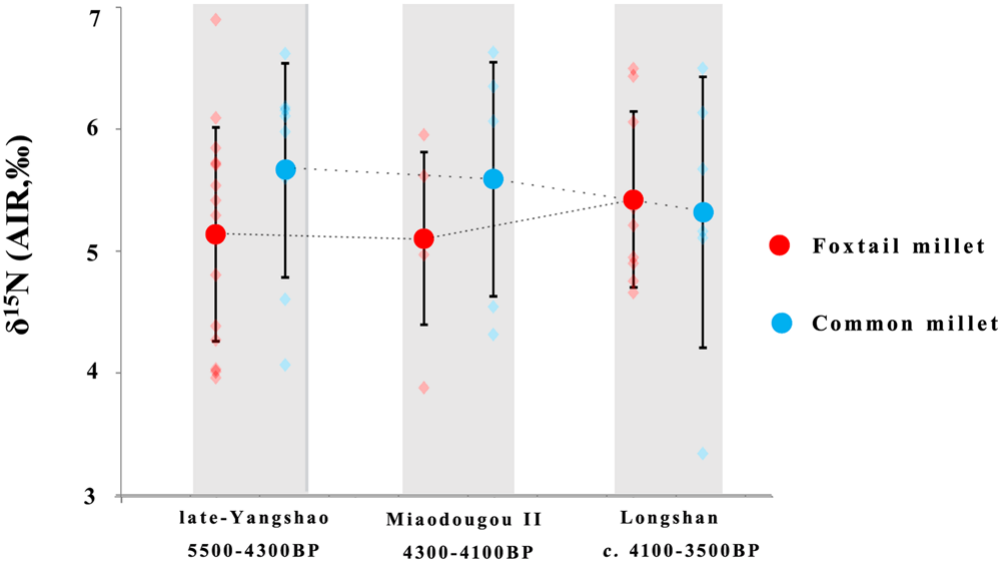
δ^15^N values of the foxtail and common millet grains grouped according to the archaeological time periods.

The variation in the δ^15^N values of the millets are plotted according to the archaeological phases in Fig. 3 to examine the variation with time. During the late-Yangshao culture, the mean δ^15^N value of the foxtail millet is 5.1 ± 0.9‰ (n = 15) and that of the common millet is 5.7 ± 0.9‰ (n = 8). During the Miaodigou II period, the mean δ^15^N value of the foxtail and common millet is 5.1 ± 0.8‰ (n = 5) and 5.6 ± 1.0‰ (n = 6), respectively, similar to that during the late-Yangshao culture. During the Longshan culture the mean ± SD δ^15^N values of the foxtail millet (5.4 ± 0.7‰, n = 9) and common millet (5.3 ± 1.1‰, n = 6) are similar. The mean δ^15^N values of the foxtail and common millet stay between ~5–6‰ and the standard deviations overlap, which indicates consistent environmental and/or agricultural trends during these three intervals of time.

### Faunal isotopic results

**Figure 4:**
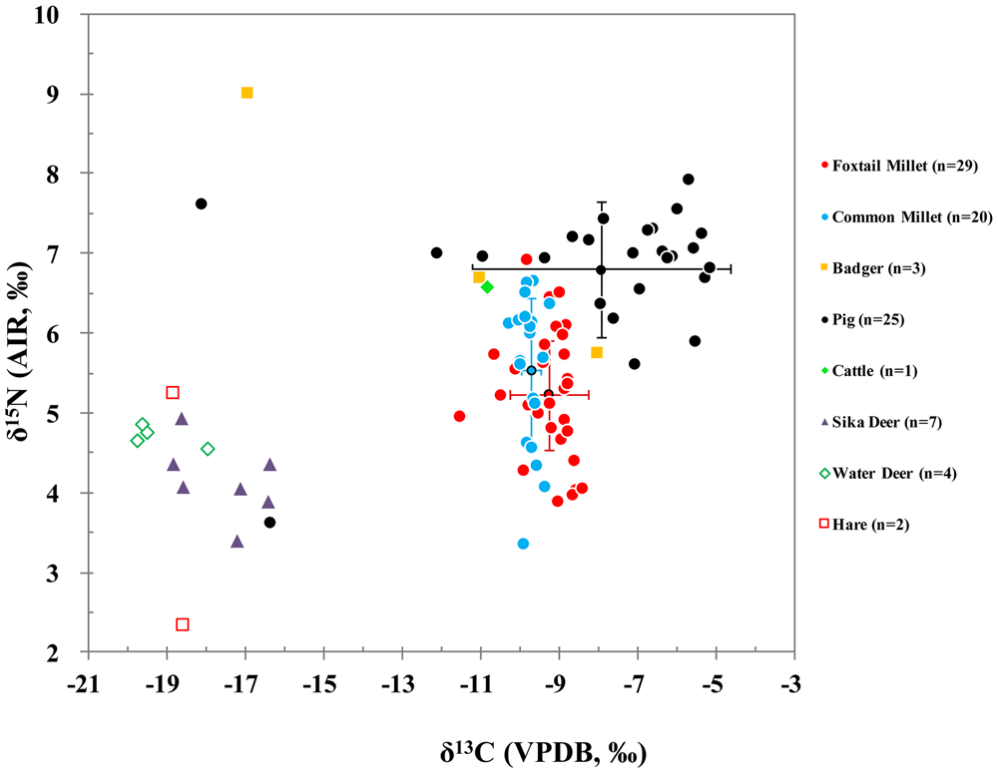
δ^13^C and δ^15^N results from millet grains and animal bone collagen from late Neolithic sites located in the Baishui River Valley.

The δ^13^C and δ^15^N values of the pigs (*Sus domesticus*, n = 25), sika deer (*Cerves nippon*, n = 7), water deer (*Hydropotes inermis*, n = 4), badgers (*Meles meles*, n = 3), hare (*Lepus capensis*, n = 2) and a single head of cattle (*Bos taurus*, n = 1) are shown in Fig. 4, and the corresponding results are summarized in Table S3. The fauna can be divided into wild and domestic animals, each showing different isotopic values. As wild herbivores, the sika and water deer and the hares show the lowest isotopic values; indicative of a diet composed of mainly C_3_ terrestrial plants (Fig. 4). The water deer have the lowest δ^13^C values ranging from −19.8‰ to −18.0‰ with a mean of −19.2 ± 0.8‰. Compared to the above, the δ^13^C values of the hare are a little higher with a mean of −18.7‰. The sika deer have the highest δ^13^C values, with a mean of −17.6 ± 1.1‰, suggesting that they might have consumed some C_4_ plants. In general, the δ^15^N values of these three animals are similar in Fig. 4 and have a mean of 4.3 ± 0.8‰, which can be used as the isotopic baseline of the natural environment (see below). The three badgers have higher δ^13^C and δ^15^N values than the above herbivores. However, their large δ^13^C and δ^15^N variations, show that their diets were diverse, ranging from C_3_/C_4_-based diets to C_4_-based diets. The domestic animals (cattle and twenty-five pigs) have higher δ^13^C and δ^15^N values than the natural herbivores. The δ^13^C value of the cattle (−10.9‰) shows that it consumed a large quantity of C_4_ plants as its fodder. The pigs represent the largest isotopic dataset in this study. Most of the pigs (except for two outliers) have high δ^13^C values and have a mean of −7.1 ± 1.8‰, indicating that they were fed mainly C_4_-based foods (millets).

## Discussion

**Figure 5:**
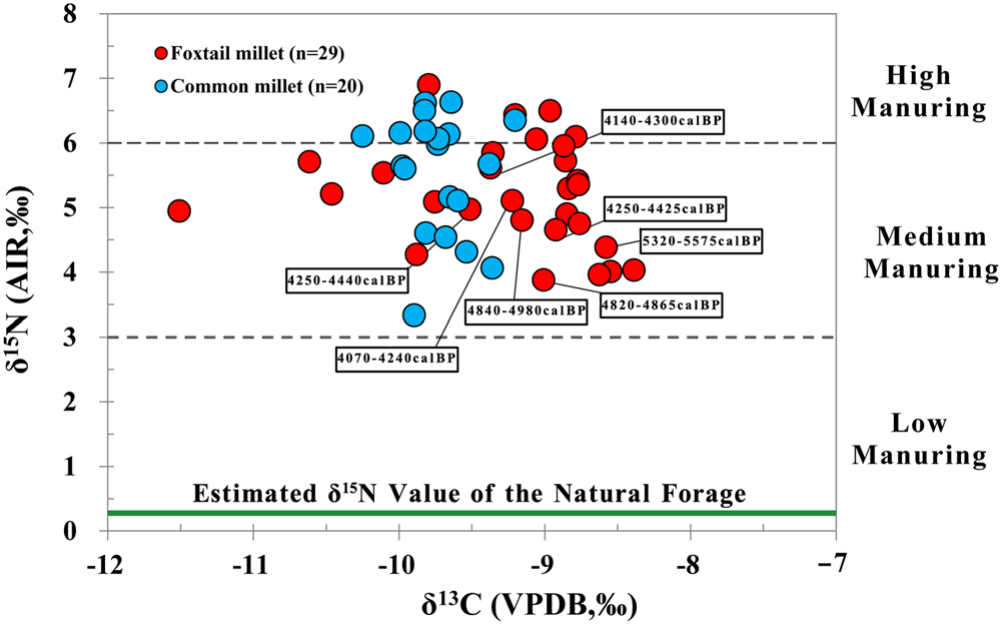
Foxtail and common millet isotopic results and radiocarbon ages from Late Neolithic sites located in the Baishui Valley graphed in relation to the estimated amount of manuring from European archaeological sites; δ^15^N values: > 6‰ represent high manuring, 3‰ to 6‰ represent medium manuring and <3% represent low manuring^8^.

When grown under uniform conditions, different plants generally display similar δ^15^N values, in spite of subtle variations among species^37^. However, the δ^15^N values of crops can be variable as a result of different human management practices such as irrigation, manuring, crop rotation, etc.^38^. Thus, it is important to determine the isotopic baseline of the local vegetation before considering possible anthropogenic effects on the soils and crops. While wild plant remains can be found with cultivated species, there can be ambiguity as to whether or not these wild plants also grew in the arable fields, and as a result it has become routine to calculate the δ^15^N values of the natural vegetation based on the isotopic data of wild herbivores^8,16,39,40^. Here the mean δ^15^N value of the natural vegetation is estimated to be equal to 0.3‰, and this was calculated by subtracting 4‰ (the mean isotopic trophic level increase)^41^ from the mean δ^15^N value (4.3 ± 0.8‰, n = 13) of the wild herbivores. Therefore, the wild plants growing in the natural environment should have values near 0‰ (Fig. 5). However, we should point out the fact that the isotopic values of the wild plants reconstructed here only represent the plants (leaves, grasses) consumed by the herbivores and not all of the wild plants in the natural vegetation. In addition, the isotopic baseline for C_4_ plants, including foxtail and common millet, that have been grown in natural (non-manured) soils is unknown. Therefore, this calculation represents a rough estimation of the δ^15^N values of the natural vegetation without anthropogenic manuring practices.

Figure 5 illustrates the δ^13^C and δ^15^N values of the millets with the solid green line representing the approximate δ^15^N value of the local vegetation. It can be seen that all the millets have higher δ^15^N values than those of the local vegetation, and this strongly suggests that the millets from the Baishui River Valley were grown with animal manure. From the associated animal data (Fig. 4), the most likely source of this manure was the domestic pigs, since these are the most numerous faunal remains recovered at the Xiahe and Mapo sites, and they have similar isotopic results to the millets. In addition, domestic cattle were also another possible source of manure.

**Figure 6:**
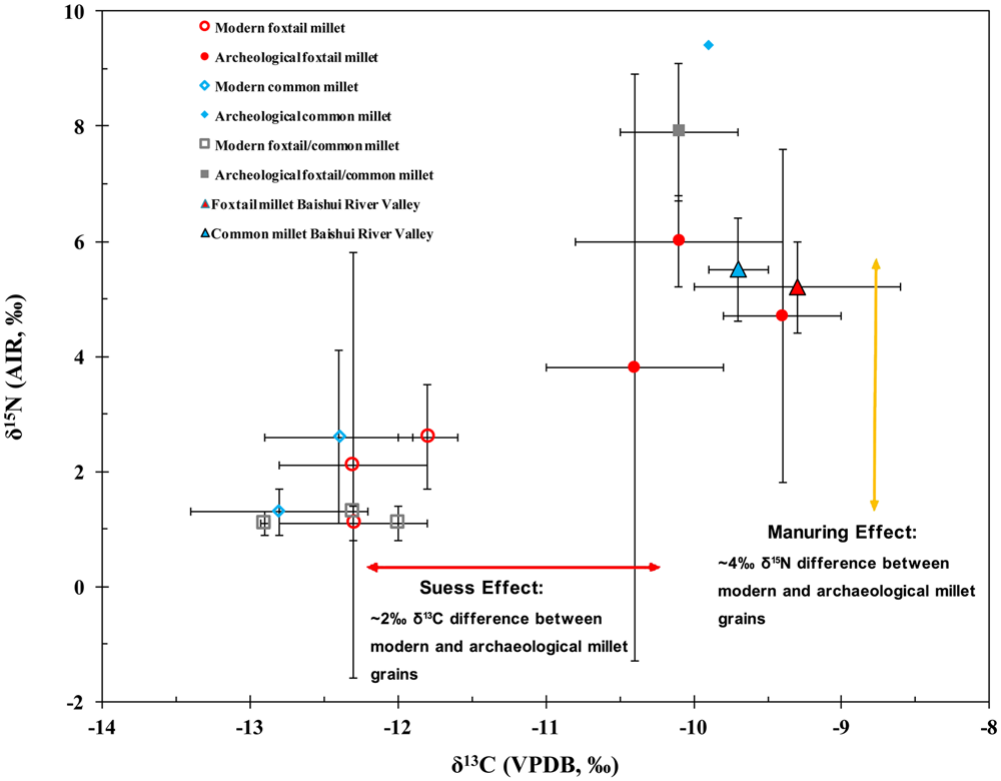
Plot of modern and archaeological δ^13^C and δ^15^N values of foxtail and common millets from China. See Table S4 for additional information and references.

The δ^13^C and δ^15^N results of the foxtail and common millets from the Baishui River Valley are plotted in relation to the previously published archaeological and modern millet results from China in Fig. 6. Unfortunately, these modern studies did not include information about the soil or if organic and/or chemical fertilizer were used. However, it is reasonable to infer that these millets might have been grown on natural soils or with the addition of chemical fertilizers given their low δ^15^N values. All of the Chinese archeological millets show ^13^C- and ^15^N-enriched values compared to the modern millets. The δ^13^C offset of ~2‰ between modern and archaeological millets is likely the result of the Suess effect or the introduction of isotopically light carbon to the atmosphere as a result of anthropogenic activity^42^. In contrast, the ~4‰ δ^15^N offset between the modern and archeological Chinese millets appears to be the result of the manuring effect (Fig. 6)^8^.

Currently, no modern plant experiments have investigated how the isotopic effects of millet manuring correlate with the degree or amount of manure added to the soil. Thus, we must rely on the isotopic model designed for wheat and barley^8^, in order to estimate the amount of manure that may have been added to the millet. According to the above model, cutoffs of <3‰, 3‰ to 6‰ and >6‰ are respectively used to discern the effects of low (~no manure), medium (10–15 tons/ha) and high manuring (35+ tons/ha)^8^. At the Baishui River Valley sites all of the foxtail and common millet isotopic results are above 3‰, possibly indicative of at least medium amounts of manuring, and 36% of the millet grain have δ^15^N values greater than 6‰, which is suggestive of high amounts of manuring (Fig. 6; Table S2). Further, the fact that the millet δ^15^N values continue to stay elevated from 5575 to 4070 cal BP indicates that there was long-term human management of the arable soils through the addition of manure in the Baishui River Valley (Figs 3 and 5).

It should be noted that recent isotopic research in the UK found modern foxtail millet with high δ^15^N values (5.1 ± 1.5‰, n = 29) as well as a large range of values (~6‰)^43^. These results were not directly related to the addition of manure but reflect the natural isotopic signatures of the soil and fertilizers used in this experiment. In contrast, nearly all the δ^15^N values of modern millets grown in China are around ~2‰ (Fig. 6 and Table S4). The reason for this isotopic difference appears to be the addition of compost to the soil in terms of the foxtail millet grown in the UK in contrast to the Chinese millets which were likely grown in natural soils and/or with chemical fertilizers. Rich in organic matter and micro-organisms, compost is more ^15^N-enriched than natural soil^38,44^ and will have its own ‘manuring effect’ and cause elevated δ^15^N values in a millet plant. In addition, the common millets which were grown in the same UK laboratory but originally from China show more positive δ^15^N values than those grown in China^45^. Thus, it is reasonable to infer that the δ^15^N values of the non-manured millets are much lower in China.

Loess are terrestrial deposits formed during the Quaternary, which contains large amount of silt particles and micaceous minerals^46^. Loess soils are regarded as one of the most fertile soils for agriculture due to its unique soil properties and nutrients^46^. However, one of the greatest disadvantage of loess soils is that they contain little clay, which makes the loss of organic matter a serious concern for these soils, and can result in poor crop germination and diminished yields over time^46^. Thus, the nitrogen content of loess soils are considered too low to be intensively cultivated^46^. However, organic manuring or the addition of animal dung to these soils can dramatically alter the soil conditions and improve the soil fertility and maintain the production of crops^47–49^. Further, cultivation experiments using wheat and millets show that the long-term organic manuring of loess soils can improve the carbon and nitrogen contents in these soils, increase the root growth and the activity of soil enzymes around the roots, stimulate the growth and development of the crops and enhance the production of the crop yields and qualities^50–53^. In particular, one of the most important measures for improving the modern production of millets in loess soils is the addition of manure through organic fertilizers^54,55^. However, additional studies are needed to better understand how the isotopic results of millet grains are directly influenced by changing soil and environmental conditions as well as the addition of different quantities of manure so that these effects can be modelled in greater detail.

A wetter and warmer climate was found to occur during the mid-Holocene (8500-3100 BP) on the Loess Plateau of China^56^, and these conditions helped created the necessary environment for the Neolithic farmers to popularize the cultivation of millets in loess soils. The isotopic evidence from numerous sites in the Loess Plateau^57–62^, including the sites studied here, show that humans and domestic animals, such as pigs, dogs, cattle and sheep relied heavily on millet agriculture during the Neolithic. While the practice of slash-and-burn agriculture could have been practiced as a means for expanding cultivation areas to increase crop yields to meet the pressures of human population growth, the loess soil fertility would quickly decrease after cultivation for many years. Thus, it appears highly unlikely that the long-term cultivation and expansion of millet agriculture could have been sustained on the Loess Plateau without the use of organic fertilizer (manuring).

Archaeozoological and isotopic studies indicate that pig domestication was highly advanced after the Yangshao culture period and that domestic pigs were a significant contributor to human diets and one of the most frequent domestic animals recovered at Neolithic sites^63–65^. At most Yangshao and Longshan period sites, pigs were found to have had diets mainly composed of millets^57,58^ and animal pens were excavated at the Banpo site which is close to the Baishui River Valley^66^. Here at the Baishui River sites in this paper, the mean δ^15^N offset between the millet grains and the pigs is only 1.3‰, much smaller than expected if the pigs were mainly eating the grains of the millets. Research on modern foxtail millet has shown that grain δ^15^N values are approximately 2‰ higher than leaves^33,43^. This is possible evidence that the pigs of the Baishui River Valley were foddered mostly on millet leaves or other byproducts and were not fed significant amounts of grains, which would have been reserved for human consumption^67^.

Domestic pigs fed by human leftovers or millet byproducts could produce large quantity of feces, which is an ideal substrate for the organic manuring of loess soils. For example, the yield of modern millets that are manured by pig dung can increase by ~18%, and pig dung is considered as the best organic fertilizer of all the domestic animals^53^. Therefore, the large quantities of pig dung created as a result of this intensive use of domestic pigs during the Late Neolithic, was collected and used as organic fertilizer to improve soil fertility and crop production. From this perspective, the practice of organically manuring millets can be viewed an important driving factor to account for the expansion of millet agriculture and population growth during the Late Neolithic of north China.

## Methods

Intact grains of charred foxtail and common millet grains were selected for isotopic analysis and radiocarbon dating from the seven sites in the Baishui Valley (Fig. 1). A total of 49 bulk millet measurements consisting of 29 foxtail and 20 common millet samples were analyzed for δ^13^C and δ^15^N values (Tables S1, S2). The bulk measurements were determined with 15 individual grains for foxtail millet and 5 individual grains for common millet, all from the same archaeological context, in order to achieve the optimal mass required for isotopic analysis. Whole and undistorted grains were selected and all visible surface contaminants were removed under microscope (Fig. 1C). Faunal remains (n = 42) were identified by Songmei Hu from the Xiahe and Mapo sites and consisted of: pigs (*Sus domesticus*, n = 25), sika deer (*Cerves nippon*, n = 7), water deer (*Hydropotes inermis*, n = 4), badgers (*Meles meles*, n = 3), hare (*Lepus capensis*, n = 2) and a single head of cattle (*Bos taurus*, n = 1) (Table S3).

The archaeological millet samples were pretreated based on a modified method of Vaiglova *et al*.^68^, but no pre-screening procedure using FTIR was used here given the good preservation of the seeds. This method involved the treatment of the millet grains with 0.5 M HCl at 80 °C for 30 min (or until effervescence stopped) followed by three rinses in distilled water. Dried grains were crushed to homogeneous powder in an agate mortar/pestle and weighed into tin containers (0.2–0.4 mg) for isotopic measurement. Bone collagen was isolated at the Key Laboratory of Vertebrate Evolution and Human Origins of Chinese Academy of Sciences, Institute of Vertebrate Palaeontology and Palaeoanthropology, Chinese Academy of Sciences using the method described by Richards and Hedges^5^, and measured at the Department of Archaeology and Anthropology at the University of the Chinese Academy of Sciences. The mass spectrometer used was an IsoPrime 100 IRMS (Elementar, UK) coupled with an ElementarVario (Elementar, UK). Isotope ratios (^13^C/^12^C or ^15^N/^14^N) are expressed as δ in per mil (‰) relative to the internationally defined standards for carbon (Vienna Pee Dee Belemnite, VPDB) and nitrogen (Ambient Inhalable Reservoir, AIR). The standards were Sulfanilamide, IAEA-600, IEAE-N-1, IAEA-N-2, IAEA-CH-6, USGS-24, USGS 40 and USGS 41 and for every 10 samples, a collagen lab standard (δ^13^C value of 14.7 ± 0.2‰ and δ^15^N value of 6.9 ± 0.2‰) was also inserted in the run for isotopic calibration. The measurement errors were less than ± 0.2‰ for both δ^13^C and δ^15^N values. No isotopic correction factors were applied to those charred seeds, as there are only minor isotopic changes of δ^13^C and δ^15^N values of seeds after charring^69–71^. All fauna specimens produced excellent collagen yields between 1.2% and 16.6% and C:N between 2.9 to 3.3, indicating that the collagen was well-preserved and acceptable for isotopic analysis (Table S3)^72^

## Electronic supplementary material

Supplementary Information
