# Supplementary material for: Millet manuring as a driving force for the Late Neolithic agricultural expansion of north China
Source: Sci Rep. 2018 Apr 3;8:5552. doi: 10.1038/s41598-018-23315-4 (PMC5882897; doi:10.1038/s41598-018-23315-4)
Supplement: Supplementary file 1 — Supplementary Information [file 41598_2018_23315_MOESM1_ESM.doc]

Supplementary Information:

**Millet manuring as a driving force for the Late Neolithic agricultural expansion of north China**

Xin Wang^1,2^, Benjamin T. Fuller^2,3^, Pengcheng Zhang^4^, Songmei Hu^4^, Yaowu Hu^1,2^*, Xue Shang^1,2^*

*^1^Key Laboratory of Vertebrate Evolution and Human Origins of Chinese Academy of Sciences, Institute of Vertebrate Paleontology and Paleoanthropology, Chinese Academy of Sciences, Beijing 100044, China*

*^2^**Department of Archaeology and Anthropology, University of Chinese Academy of Sciences, Beijing 100049, China*

*^3^Department of Archaeology and Heritage Studies, School of Culture and Society, Aarhus University, Moesgård Allé 20, DK-8270, Højbjerg, Denmark*

*^4^Shaanxi Provincial Institute of Archaeology, Xi’an 710043, China*

*Correspondence to:

Yaowu Hu

Department of Archeology and Anthropology

University of Chinese Academy of Sciences

19A, Yuquan Road

Beijing 100049, China

Tel: +8618518095070

E-mail: [ywhu@ucas.ac.cn](mailto:ywhu@ucas.ac.cn)

Xue Shang

Department of Archeology and Anthropology

University of Chinese Academy of Sciences

19A, Yuquan Road

Beijing 100049, China

Tel: +8618501255223

E-mail: [shangxue@ucas.ac.cn](mailto:shangxue@ucas.ac.cn)

This Supplementary Text file includes:

Stable isotope ratio analysis

Tables S1 to S4

References

**Stable isotope ratio analysis**

Carbon (δ^13^C) and nitrogen (δ^15^N) stable isotope ratio analysis is a routine and established method to investigate the subsistence patterns of ancient humans and animals[^1-9^](#_ENREF_1). Measurement of δ^13^C values can be used to identify carbon derived from C_3_ plants (those adapted to temperate climates; most vegetables, fruit, and grains) vs. carbon from C_4_ plants (those adapted to hot and arid climates; most importantly for China, millets)[^10-12^](#_ENREF_10). This is due to the fact that C_3_ plants show increased ^13^C-discrimination when fixing atmospheric CO_2_ for tissue synthesis. Thus, C_3_ plants have more negative δ^13^C results (–30‰ to –22‰) compared to C_4_ plants (–16‰ to –10‰), with mean values of approximately –26.5‰ and –12.5‰, respectively[^13-15^](#_ENREF_13). These large isotopic differences between C_3_ and C_4_ plant species can be used as markers to investigate the importance of millets for human and animal diets at archaeological sites in China.

The analysis of δ^15^N values permits an investigation of protein consumption and trophic level position, due to a stepwise ^15^N-enrichment of 3-5‰ from diet to consumer in the food chain[^18-22^](#_ENREF_18). Of special note for isotopic studies focused on plants, δ^15^N results are also influenced by the type of nitrogen fixation (non-N_2_-fixation vs. N_2_-fixation plants), climate and soil conditions (i.e. temperature, rainfall, aridity, salinity), manuring effects and species variation. For additional information about the uses and applications of stable isotope ratios in archaeology see the following publications .

The isotopic data of modern millet grains published were listed in Table S4, which will be used as a comparison to understand the manuring effects in the past. In general, the δ^15^N values of those modern millets are low and range from 1‰ ~ 3‰. Unfortunately, in the previously published papers the isotopic data of the modern millets did not include the necessary information on the soil properties and fertilizers used. However, it is reasonable to infer that those millets might have been grown naturally or with the use of chemical fertilizers on the basis of their low δ^15^N values.

Table S1. Details of the Baishui Valley sites, Shaanxi Province, China.

| **Site** | **Location (latitude N, longitude E)** | **Relative chronology** | **Absolute date range (BP)** | ***n* Crop** | ***n* Animal** | **Geological information** |
| --- | --- | --- | --- | --- | --- | --- |
| **Xiahe** | 35.16,109.63 | Late Yangshao-Longshan | 5190-3500 | 30 | 23 | Mean annual temperature: 11.4℃,  Annual average precipitation: 577.8mm, [^39^](#_ENREF_39)  Soil organic matter content: 0.95%,  Nitrogen content: 0.067% [^40^](#_ENREF_40) |
| **Mapo** | 35.14,109.60 | Miaodigou II | 4300-4140 | 1 | 14 |
| **Nanshantou** | 35.18,109.57 | Yangshao-Longshan | 5480-3500 | 5 |  |
| **Beishantou** | 35.18,109.56 | Late Yangshao | 5750-5320 | 6 |  |
| **Hanzhai** | 35.26,109.52 | Miaodigou II&Longshan | 4440-4250 | 4 |  |
| **Xishan** | 35.16,109.63 | Late Yangshao | 4980-4820 | 1 |  |
| **Muwanghe** | 35.14,109.69 | Early Yangshao | 5480-5310 | 2 |  |

Table S2. Sample information and isotopic results for the foxtail and common millet grains from the Baishui Valley sites, Shaanxi Province, China.

| No. | Taxon | Culture | Site | Unit | Quantity | Weight (mg) | δ^13^C | δ^15^N | (%)C | (%)N | Atomic C:N |
| --- | --- | --- | --- | --- | --- | --- | --- | --- | --- | --- | --- |
| 1 | Foxtail millet | Late Yangshao | Xiahe | G1① | 15 | 2.74 | -8.9 | 5.7 | 57.8 | 3.8 | 17.6 |
| 2 | Foxtail millet | Late Yangshao | Xiahe | XH06 | 15 | 3.40 | -10.1 | 5.5 | 60.1 | 4.3 | 16.2 |
| 3 | Foxtail millet | Late Yangshao | Xiahe | XH08 | 15 | 2.82 | -8.8 | 5.3 | 60.4 | 3.6 | 19.4 |
| 4 | Foxtail millet | Late Yangshao | Xiahe | XH09 | 15 | 3.06 | -10.6 | 5.7 | 56.2 | 3.8 | 17.4 |
| 5 | Foxtail millet | Late Yangshao | Xiahe | H9 | 15 | 3.11 | -8.8 | 6.1 | 57.5 | 4.2 | 16.2 |
| 6 | Foxtail millet | Late Yangshao | Xiahe | G1③ | 15 | 2.91 | -9.8 | 6.9 | 44.1 | 4.3 | 12.1 |
| 7 | Foxtail millet | Late Yangshao | Xiahe | G1 | 15 | 2.74 | -8.8 | 5.4 | 57.6 | 3.9 | 17.4 |
| 8 | Foxtail millet | Late Yangshao | Xiahe | H48 | 15 | 3.70 | -9.8 | 5.1 | 60.2 | 3.5 | 19.9 |
| 9 | Foxtail millet | Miaodigou II | Xiahe | H20② | 15 | 3.11 | -9.2 | 5.1 | 61.4 | 4.5 | 16.1 |
| 10 | Foxtail millet | Longshan | Xiahe | T2⑤ | 15 | 4.26 | -8.8 | 4.9 | 62.6 | 3.7 | 19.9 |
| 11 | Foxtail millet | Longshan | Xiahe | T2⑤ | 15 | 2.33 | -8.8 | 5.4 | 60.3 | 3.6 | 19.8 |
| 12 | Foxtail millet | Longshan | Xiahe | H45 | 15 | 3.20 | -8.9 | 4.7 | 61.0 | 3.8 | 18.8 |
| 13 | Foxtail millet | Longshan | Xiahe | H24① | 15 | 2.83 | -9.2 | 6.4 | 59.7 | 4.2 | 16.7 |
| 14 | Foxtail millet | Longshan | Xiahe | H24② | 15 | 2.10 | -10.5 | 5.2 | 59.7 | 4.2 | 16.5 |
| 15 | Foxtail millet | Longshan | Xiahe | H19 | 15 | 3.14 | -8.8 | 4.8 | 61.4 | 3.5 | 20.5 |
| 16 | Foxtail millet | Longshan | Xiahe | H34 | 15 | 3.20 | -9.1 | 6.1 | 59.5 | 4.8 | 14.5 |
| 17 | Foxtail millet | Miaodigou II | Xiahe | H20② | 20 | 4.1 | -8.9 | 6.0 | 63.8 | 4.2 | 13.0 |
| 18 | Foxtail millet | Early Yangshao | Nanshantou | H15 | 15 | 2.22 | -9.2 | 4.8 | 77.5 | 4.9 | 18.5 |
| 19 | Foxtail millet | Miaodigou II | Nanshantou | H10 | 15 | 3.01 | -9.0 | 3.9 | 61.3 | 4.7 | 15.1 |
| 20 | Foxtail millet | Longshan | Nanshantou | H12 | 15 | 1.83 | -11.5 | 4.9 | 58.8 | 4.4 | 15.6 |
| 21 | Foxtail millet | Miaodigou II | Mapo | H1 | 15 | 2.68 | -9.4 | 5.6 | 59.1 | 5.2 | 13.3 |
| 22 | Foxtail millet | Longshan | Hanzhai | H10 | 15 | 3.47 | -9.0 | 6.5 | 59.9 | 4.6 | 15.4 |
| 23 | Foxtail millet | Miaodigou II | Hanzhai | H2 | 15 | 3.34 | -9.5 | 5.0 | 60.2 | 5.2 | 13.5 |
| 24 | Foxtail millet | Late Yangshao | Xishan | H2 | 15 | 1.98 | -9.4 | 5.8 | 59.6 | 4.3 | 16.1 |
| 25 | Foxtail millet | Early Yangshao | Beishantou | H1 | 15 | 4.3 | -9.9 | 4.3 | 48.4 | 2.7 | 20.6 |
| 26 | Foxtail millet | Early Yangshao | Beishantou | H1 | 15 | 4.4 | -8.5 | 4.0 | 63.8 | 4.1 | 18.3 |
| 27 | Foxtail millet | Early Yangshao | Beishantou | H1 | 20 | 3.2 | -8.6 | 4.0 | 64.5 | 4.1 | 18.5 |
| 28 | Foxtail millet | Early Yangshao | Beishantou | H1 | 15 | 2.3 | -8.4 | 4.0 | 63.7 | 4.1 | 18.2 |
| 29 | Foxtail millet | Early Yangshao | Beishantou | H1 | 20 | 3.8 | -8.6 | 4.4 | 63.8 | 4.1 | 18.3 |
| 30 | Common millet | Late Yangshan | Xiahe | G1 | 5 | 2.14 | -9.7 | 6.0 | 58.7 | 3.1 | 22.4 |
| 31 | Common millet | Late Yangshan | Xiahe | XH08 | 5 | 2.71 | -10.3 | 6.1 | 60.7 | 3.6 | 19.7 |
| 32 | Common millet | Late Yangshan | Xiahe | H9 | 5 | 2.92 | -10.0 | 6.2 | 58.0 | 3.3 | 20.5 |
| 33 | Common millet | Late Yangshan | Xiahe | H48 | 5 | 3.44 | -9.8 | 4.6 | 58.9 | 4.6 | 14.8 |
| 34 | Common millet | Miaodigou II | Xiahe | H20② | 5 | 3.44 | -9.2 | 6.3 | 60.8 | 5.3 | 13.3 |
| 35 | Common millet | Longshan | Xiahe | H45 | 5 | 2.23 | -9.7 | 5.2 | 60.3 | 3.8 | 18.8 |
| 36 | Common millet | Longshan | Xiahe | H19 | 5 | 1.47 | -9.9 | 3.3 | 61.5 | 3.1 | 23.1 |
| 37 | Common millet | Longshan | Xiahe | H34 | 5 | 1.75 | -9.4 | 5.7 | 60.8 | 3.5 | 20.6 |
| 38 | Common millet | Longshan | Xiahe | H24② | 5 | 1.77 | -9.6 | 5.1 | 60.5 | 4.0 | 17.6 |
| 39 | Common millet | Miaodigou II | Xiahe | H20② | 6 | 3.3 | -9.7 | 4.5 | 63.5 | 3.9 | 14.0 |
| 40 | Common millet | Miaodigou II | Xiahe | H20② | 6 | 3.1 | -10.0 | 5.6 | 61.6 | 4.4 | 12.0 |
| 41 | Common millet | Miaodigou II | Xiahe | H20② | 6 | 3.3 | -9.5 | 4.3 | 63.2 | 4.1 | 13.3 |
| 42 | Common millet | Miaodigou II | Xiahe | H20② | 6 | 3.5 | -9.6 | 6.6 | 8.5 | 2.0 | 3.7 |
| 43 | Common millet | Early Yangshao | Nanshantou | H15 | 5 | 2.08 | -9.8 | 6.6 | 59.8 | 4.7 | 14.9 |
| 44 | Common millet | Longshan | Nanshantou | H11 | 5 | 1.14 | -9.7 | 6.1 | 61.0 | 4.2 | 17.0 |
| 45 | Common millet | Miaodigou II | Hanzhai | H2 | 5 | 2.97 | -9.7 | 6.1 | 57.7 | 5.0 | 13.4 |
| 46 | Common millet | Longshan | Hanzhai | H10 | 5 | 3.07 | -9.8 | 6.5 | 62.1 | 4.0 | 18.3 |
| 47 | Common millet | Early Yangshao | Beishantou | H1 | 20 | 3.5 | -9.4 | 4.1 | 62.9 | 3.6 | 20.4 |
| 48 | Common millet | Early Yangshao | Muwanghe | H1 | 6 | 3.6 | -9.8 | 6.2 | 61.3 | 3.7 | 19.2 |
| 49 | Common millet | Early Yangshao | Muwanghe | H1 | 10 | 3.4 | -10.0 | 5.6 | 61.1 | 4.2 | 17.1 |

Table S3. Sample information and isotopic results for the animals from the Baishui Valley sites, Shaanxi Province, China.

| No. | Taxon | Element | Site | Unit | % Collagen yield | δ^13^C | δ^15^N | (%)C | (%)N | Atomic C:N |
| --- | --- | --- | --- | --- | --- | --- | --- | --- | --- | --- |
| B1 | Pig | Humerus | Xiahe | H15:17 | 7.1 | -18.1 | 7.6 | 44.0 | 15.3 | 3.3 |
| B2 | Pig | Humerus | Xiahe | H15:21 | 5.6 | -5.3 | 7.2 | 42.5 | 15.1 | 3.3 |
| B3 | Pig | Femur | Xiahe | H15:26 | 3.9 | -6.6 | 7.3 | 43.9 | 15.5 | 3.3 |
| B4 | Pig | Tibia | Xiahe | H26:3 | 3.7 | -7.8 | 7.4 | 43.7 | 15.3 | 3.3 |
| B5 | Pig | Humerus | Xiahe | H16:10 | 9.5 | -5.6 | 7.0 | 44.8 | 15.7 | 3.3 |
| B6 | Pig | Humerus | Xiahe | T0208④:16 | 3.3 | -6.1 | 6.9 | 42.6 | 15.2 | 3.3 |
| B7 | Pig | Radius | Xiahe | H16:11 | 9.9 | -5.3 | 6.7 | 40.8 | 14.8 | 3.2 |
| B8 | Pig | Humerus | Xiahe | H7:41 | 7.1 | -6.3 | 7.0 | 44.3 | 15.9 | 3.2 |
| B9 | Pig | Humerus | Xiahe | H7:39 | 6.5 | -5.7 | 7.9 | 44.4 | 16.0 | 3.2 |
| B10 | Pig | Radius | Xiahe | H45:101 | 8.7 | -16.3 | 3.6 | 44.0 | 15.9 | 3.2 |
| B11 | Pig | Femur | Xiahe | F1Z1:590 | 4.3 | -6.0 | 7.5 | 44.4 | 16.1 | 3.2 |
| B12 | Pig | Tibia | Xiahe | F1Z1:607 | 1.2 | -6.7 | 7.3 | 36.2 | 13.6 | 3.1 |
| B13 | Pig | Tibia | Xiahe | F1Z1:609 | 15.1 | -9.3 | 6.9 | 42.7 | 15.6 | 3.2 |
| B14 | Pig | Tibia | Xiahe | H52:3 | 6.9 | -10.9 | 6.9 | 44.0 | 15.9 | 3.2 |
| B15 | Pig | Tibia | Xiahe | H52:4 | 3.3 | -6.2 | 6.9 | 44.9 | 16.2 | 3.2 |
| B16 | Pig | Humerus | Xiahe | H7:40 | 6.8 | -5.2 | 6.8 | 37.9 | 13.7 | 3.2 |
| B17 | Pig | Underjaw | Mapo | H1:26 | 16.6 | -6.9 | 6.5 | 45.3 | 16.2 | 3.3 |
| B18 | Pig | Cranium | Mapo | H1:14 | 13.5 | -7.1 | 5.6 | 44.8 | 16.1 | 3.2 |
| B19 | Pig | Humerus | Mapo | H1:36 | 5.5 | -7.6 | 6.2 | 43.8 | 15.7 | 3.3 |
| B20 | Pig | Humerus | Mapo | H1:34 | 5.1 | -5.5 | 5.9 | 43.8 | 15.9 | 3.2 |
| B21 | Pig | Radius | Mapo | H1:42 | 11.4 | -8.2 | 7.2 | 44.5 | 15.9 | 3.3 |
| B22 | Pig | Ulna | Mapo | H1:39 | 10.8 | -8.6 | 7.2 | 44.9 | 16.3 | 3.2 |
| B23 | Pig | Radius | Mapo | H1:41 | 2.9 | -7.9 | 6.4 | 43.8 | 16.0 | 3.2 |
| B24 | Pig | Tibia | Mapo | H1:54 | 7.1 | -12.1 | 7.0 | 43.8 | 15.6 | 3.3 |
| B25 | Pig | Ulna | Mapo | H1:38 | 7.8 | -7.1 | 7.0 | 45.8 | 16.3 | 3.3 |
| B26 | Sika Deer | Tibia | Xiahe | H45:105 | 6.3 | -17.2 | 3.4 | 44.0 | 16.2 | 3.2 |
| B27 | Sika Deer | Tibia | Xiahe | H44:45 | 7.0 | -16.4 | 3.9 | 16.0 | 5.9 | 3.2 |
| B28 | Sika Deer | Tibia | Xiahe | H45:104 | 4.1 | -16.4 | 4.4 | 42.5 | 15.3 | 3.2 |
| B29 | Sika Deer | Femur | Xiahe | H44:44 | 6.0 | -18.6 | 4.1 | 43.4 | 15.6 | 3.2 |
| B30 | Sika Deer | Tibia | Xiahe | H45:106 | 7.4 | -18.8 | 4.4 | 41.6 | 15.1 | 3.2 |
| B31 | Sika Deer | Radius | Xiahe | H45:100 | 9.5 | -17.1 | 4.0 | 38.7 | 14.6 | 3.1 |
| B32 | Sika Deer | Pelvis | Mapo | H1:88 | 12.5 | -18.6 | 4.9 | 43.8 | 15.9 | 3.2 |
| B33 | Water Deer | Humerus | Xiahe | T0512②:16 | 8.4 | -19.6 | 4.9 | 44.3 | 15.9 | 3.3 |
| B34 | Water Deer | Tibia | Xiahe | T0208⑤:59 | 6.7 | -18.0 | 4.5 | 46.4 | 16.6 | 3.3 |
| B35 | Water Deer | Tibia | Mapo | H1:73 | 4.8 | -19.8 | 4.7 | 44.3 | 16.2 | 3.2 |
| B36 | Water Deer | Humerus | Mapo | H1:70 | 7.1 | -19.5 | 4.7 | 44.3 | 16.1 | 3.2 |
| B37 | Badger | Axis | Xiahe | F1①:20 | 6.3 | -16.9 | 9.0 | 34.1 | 13.7 | 2.9 |
| B38 | Badger | Ulna | Xiahe | F1①:21 | 8.2 | -8.0 | 5.7 | 45.0 | 16.1 | 3.3 |
| B39 | Badger | Humerus | Mapo | H1:10 | 12.2 | -11.0 | 6.7 | 45.0 | 16.3 | 3.2 |
| B40 | Hare | Humerus | Xiahe | H9:4 | 6.6 | -18.6 | 2.3 | 45.1 | 16.3 | 3.2 |
| B41 | Hare | Tibia | Xiahe | H28:1 | 5.6 | -18.8 | 5.2 | 44.4 | 15.9 | 3.3 |
| B42 | Cattle | Ulna | Mapo | H1:91 | 5.0 | -10.9 | 6.6 | 44.6 | 16.2 | 3.2 |

Table S4. Summary of previously published δ^13^C and δ^15^N results for modern and archaeological foxtail and common millets in China.

| **Species** | **Location** | **Time Period** | **N** | **δ^13^C (‰)** | **±SD** | **N** | **δ^15^N (‰)** | **±SD** | **Reference** |
| --- | --- | --- | --- | --- | --- | --- | --- | --- | --- |
| Foxtail Millet | China | Modern | 4 | -11.8 | 0.2 | 4 | 2.6 | 0.9 | [31](#_ENREF_31) |
| Foxtail Millet | Loess Plateau | Modern | 13 | -12.5 | 0.4 | 0 | - | - | [32](#_ENREF_32) |
| Foxtail Millet | Loess Plateau | Modern | 22 | -12.7 | 0.5 | 0 | - | - | [33](#_ENREF_33) |
| Foxtail Millet | Loess Plateau | Modern | 14 | -12.3 | 0.5 | 14 | 2.1 | 3.7 | [16](#_ENREF_16) |
| Foxtail Millet | Loess Plateau | Modern | 66 | -12.3 | 0.5 | 66 | 1.1 | 0.3 |  |
| Foxtail Millet | Loess Plateau | 5.5-5.0 ka BP | 6 | -10.4 | 0.6 | 3 | 3.8 | 5.1 | [16](#_ENREF_16) |
| Foxtail Millet | Loess Plateau | 5.0-4.5 ka BP | 3 | -10.1 | 0.7 | 3 | 6.0 | 0.8 | [16](#_ENREF_16) |
| Foxtail Millet | Loess Plateau | 4.3-3.8 ka BP | 6 | -9.4 | 0.4 | 6 | 4.7 | 2.9 | [16](#_ENREF_16) |
| Common Millet | North China | Modern | 15 | -12.4 | 0.5 | 15 | 2.6 | 1.5 | [35](#_ENREF_35) |
| Common Millet | Loess Plateau | Modern | 10 | -13.1 | 0.5 | - | - | - | [32](#_ENREF_32) |
| Common Millet | Loess Plateau | Modern | 15 | -13.2 | 0.5 | - | - | - | [36](#_ENREF_36) |
| Common Millet | Loess Plateau | Modern | 11 | -12.8 | 0.6 | 66 | 1.3 | 0.4 |  |
| Common Millet | West China | 3.75-3.15 ka BP | 1 | -9.9 | - | 1 | 9.4 | - | [37](#_ENREF_37) |
| Common Millet | North China | 3.44-7.65 ka BP | 66 | -10.2 | 0.4 | - | - | - | [41](#_ENREF_41) |
| Foxtail/Common Millet | Heilongjiang Province | Modern | 3 | -12.0 | 0.0 | 3 | 1.1 | 0.3 | [38](#_ENREF_38) |
| Foxtail/Common Millet | Shandong Province | Modern | 3 | -12.3 | 0.0 | 3 | 1.3 | 0.2 | [38](#_ENREF_38) |
| Foxtail/Common Millet | Jiangsu Province | Modern | 3 | -12.9 | 0.0 | 3 | 1.1 | 0.2 | [38](#_ENREF_38) |
| Foxtail/Common Millet | West China | 4.15-2.85 ka BP | 3 | -10.1 | 0.4 | 3 | 7.9 | 1.2 | [37](#_ENREF_37) |

**References**
